# The First Phylogeographic Population Structure and Analysis of Transmission Dynamics of *M. africanum* West African 1— Combining Molecular Data from Benin, Nigeria and Sierra Leone

**DOI:** 10.1371/journal.pone.0077000

**Published:** 2013-10-15

**Authors:** Florian Gehre, Martin Antonio, Frank Faïhun, Mathieu Odoun, Cecile Uwizeye, Pim de Rijk, Bouke C. de Jong, Dissou Affolabi

**Affiliations:** 1 Medical Research Council (MRC) Unit, Fajara, The Gambia; 2 Institute for Tropical Medicine (ITM), Antwerp, Belgium; 3 New York University (NYU), New York, New York, United States of America; 4 Laboratoire de Reference des Mycobacteries, Cotonou, Benin; Instituto de Higiene e Medicina Tropical, Portugal

## Abstract

*Mycobacterium africanum* is an important cause of tuberculosis (TB) in West Africa. So far, two lineages called *M. africanum* West African 1 (MAF1) and *M. africanum* West African 2 (MAF2) have been defined. Although several molecular studies on MAF2 have been conducted to date, little is known about MAF1. As MAF1 is mainly present in countries around the Gulf of Guinea we aimed to estimate its prevalence in Cotonou, the biggest city in Benin. Between 2005–06 we collected strains in Cotonou/Benin and genotyped them using spoligo- and 12-loci-MIRU-VNTR-typing. Analyzing 194 isolates, we found that 31% and 6% were MAF1 and MAF2, respectively. Therefore Benin is one of the countries with the highest prevalence (37%) of *M. africanum* in general and MAF1 in particular. Moreover, we combined our data from Benin with publicly available genotyping information from Nigeria and Sierra Leone, and determined the phylogeographic population structure and genotypic clustering of MAF1. Within the MAF1 lineage, we identified an unexpected great genetic variability with the presence of at least 10 sub-lineages. Interestingly, 8 out of 10 of the discovered sub-lineages not only clustered genetically but also geographically. Besides showing a remarkable local restriction to certain regions in Benin and Nigeria, the sub-lineages differed dramatically in their capacity to transmit within the human host population. While identifying Benin as one of the countries with the highest overall prevalence of *M. africanum*, this study also contains the first detailed description of the transmission dynamics and phylogenetic composition of the MAF1 lineage.

## Introduction

*Mycobacterium africanum* is the cause of up to 40% of all tuberculosis (TB) in West Africa and can be sub-divided into two phylogenetic lineages: *M. africanum* West African 1 (MAF1) or Lineage 5, and *M. africanum* West African 2 (MAF2) or Lineage 6 [1], [2]. While MAF1 can be primarily found in Eastern West African and Central African countries, MAF2 is mainly prevalent in Western West African countries such as Guinea-Bissau and The Gambia [2]. In Sierra Leone, Ivory Coast, Ghana and Benin both lineages are found [2]. The majority of molecular and clinical studies to date were conducted on MAF2, which was associated with slower progression to active disease [3], HIV infection [4] and a less immunogenic phenotype [5]. In contrast, relatively little is known about MAF1, with the majority of epidemiological studies originating from Cameroon. While in 1971 the MAF1 prevalence (based on phenotypic identification) in Cameroon was still estimated to be 56% [6] a decrease to 9% was observed in 1997–1998 [7]. A recent report from 2013 suggested that MAF1 has almost disappeared from the country [8]. In contrast, two recent studies covering several regions of neighboring Nigeria identified a high MAF1 prevalence, ranging from 14% to 33% [9], [10], and detected active foci of recent MAF1 transmission in 2009–2010 [9]. In the present study we intended to investigate the prevalence of MAF1 and MAF2 in Benin.

We genotyped isolates collected between 2005–2006 [11] by applying spoligotyping and estimate that 37% of all TB in Cotonou, the capital of Benin, is caused by *M. africanum*. Amongst these isolates, 31% were classified as MAF1, which is one of the highest prevalence estimates of this particular mycobacterial lineage described. We also identified 6% MAF2 in the study area. By applying 12-loci MIRU-VNTR-typing to the MAF1 isolates and combining our data with published spoligotype and MIRU-VNTR data from Nigeria [9], [10] and Sierra Leone [12], we constructed a phylogeographic population structure of the MAF1 lineage. We defined several sub-lineages, some of which seemed to be locally restricted and have different capacities to transmit from patient to patient.

## Materials and Methods

### Ethics Statement

All patients gave informed consent for their information to be stored in the hospital database and used for research. The study was approved by the Ethics Board of the Faculty of Health Sciences, Abmey Calavi University, Benin.

### Strain Collection

For the current study we re-analysed strain information collected during a drug-susceptibility survey conducted in Cotonou, Benin, between July 2005 and October 2006; isolates were collected, processed and genotyped (spoligotyping and12-locus MIRU-VNTR) as described elsewhere [11]. Sampling fraction and culture positivity are also described in the original report. Briefly, out of 201 newly recruited patients, five were excluded as the culture was either negative or contaminated and no bacterial isolate was obtained. Two further isolates had indications for mixed infection and were also excluded. Therefore a total of 194 isolates were included into the present study [11]. Assignment of lineages was based on spoligotyping and 12-locus-MIRU-VNTR data and is described below. We also included previously published spoligotype and MIRU-VNTR data from 2 studies from Nigeria [9], [10] and from Sierra Leone [12].

### Analysis of Genotypic Data

For lineage assignment of each isolate based on the available spoligotyping data, we used TBLineage (http://tbinsight.cs.rpi.edu/about_tb_lineage.html) [13]. For patterns that could not be fully resolved by spoligotyping, we also considered 12-loci MIRU VNTR data. The population structure and phylogenetic trees were constructed using the MIRU-VNTRplus homepage. *Mycobacterium canetti* from the MIRUVNTRplus database was included as an outgroup (www.miru-vntrplus.org) [14], [15]. The Recent Transmission Index (RTI_n-1_) for each of the identified sub-lineages (SL) was calculated as described elsewhere [16]. Briefly, it was assumed that genotypically identical isolates belonged to the same chain-of-transmission and that each of the identified genotypic clusters contains one index case. Therefore, the proportion of recently transmitted strains within each sub-lineage is:

RTI_n-1_ = (Total number of clustered strains in SL – number of clusters in SL)\Total numbers of strains in SL.

## Results

### Prevalence of *M. africanum* in Benin

**Figure 1:**
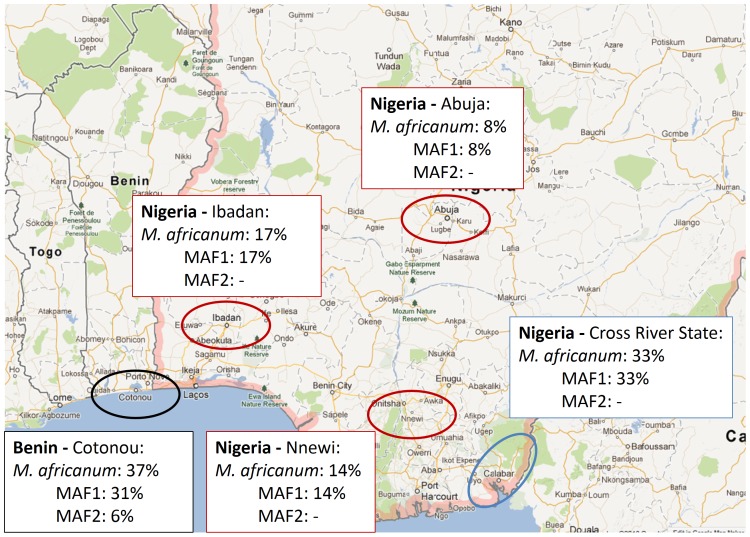
Geographical location of various study sites in Benin and Nigeria and the prevalence of *M. africanum* West Africa 1 (MAF1) and West Africa 2 (MAF2). Data from Benin was generated in the present study, the prevalence in Ibadan, Abuja and Nnewi was extracted and estimated from Lawson *et al.* [9], whereas the prevalence in the Cross River State derived from Thumamo *et al.* [10].

We genotyped 194 isolates from Benin using spoligotyping. To assign lineages we used the “TBLineage” online platform and found that 126 strains were *M. tuberculosis* which were therefore not further analyzed as we intended to focus our study on the two *M. africanum* lineages. Out of 194 spoligotyped isolates, 57 (29%) were MAF1, and 11 (6%) were MAF2 according to “TBLineage”. When confirming these results with MIRU-VNTR, two of the 57 MAF1 isolates resulted in discrepant patterns and were excluded as MAF1. Based on their spoligotype patterns another 6 isolates (3%) were correctly classified as *M. africanum*, yet “TBLineage” could not further determine whether these isolates were MAF1 or MAF2. When considering the available MIRU-VNTR data for these 6 isolates we were able to assign 5 isolates to the MAF1 lineage and one isolate to be MAF2. This resulted in a total prevalence of 31% for MAF1 and 6% for MAF2, respectively. Besides this estimate on the prevalence in Benin, data for Ibadan, Abuja, Nnewi and the Cross River State were extracted from previous publications and summarized in figure 1.

### Population Structure of *M. africanum* West African 1

**Figure 2:**
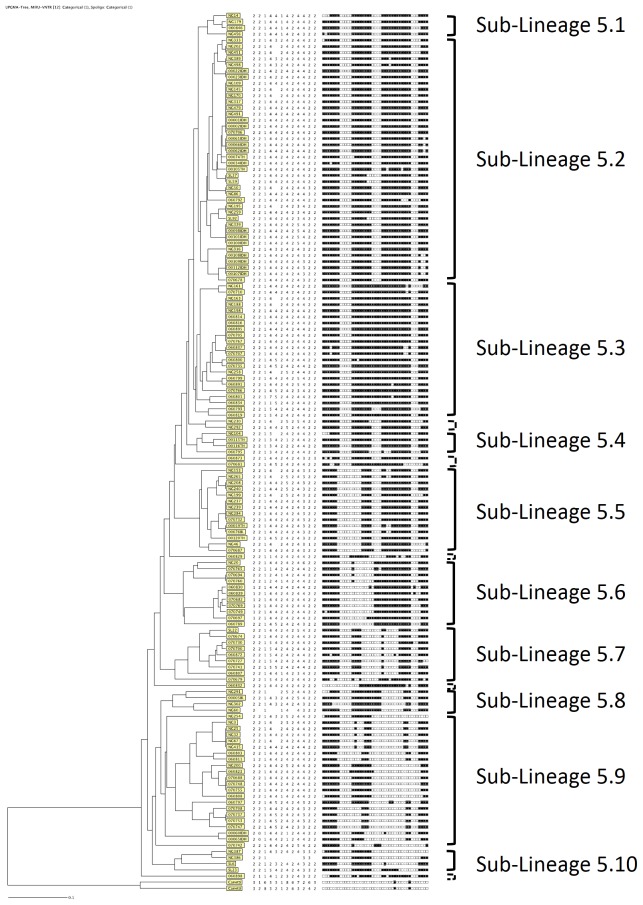
Population structure of *M. africanum* West Africa 1 (MAF1). A UPGMA tree was constructed including 141 MAF1 isolates from Benin, Sierra Leone and Nigeria. Ten sub-lineages were identified. Seven isolates could not be assigned to any of the defined sub-lineages.

We included in the population structure 60 isolates from Benin which were unambiguously identified as MAF1. Furthermore we added 81 strains that were previously described as MAF1 from recent publications and for which spoligo- and 12-loci-MIRU-VNTR typing data was publicly available. Amongst these strains from the literature were 25 isolates from the Cross River State in Nigeria [10], 50 isolates from Abuja, Ibadan and Nnewi in Nigeria [9], and 6 isolates from Sierra Leone [12]. When constructing a UPGMA tree, we identified 10 major sub-lineages (SL) within the MAF1 clade that we designated sub-lineages SL5.1–SL5.10 (see Figure 2). Moreover we found 7 isolates that could not be assigned to either of the ten sub-lineages, suggesting the presence of additional sub-lineages.

### Geographic Distribution of *M. africanum* West African 1 Sub-lineages

**Figure 3:**
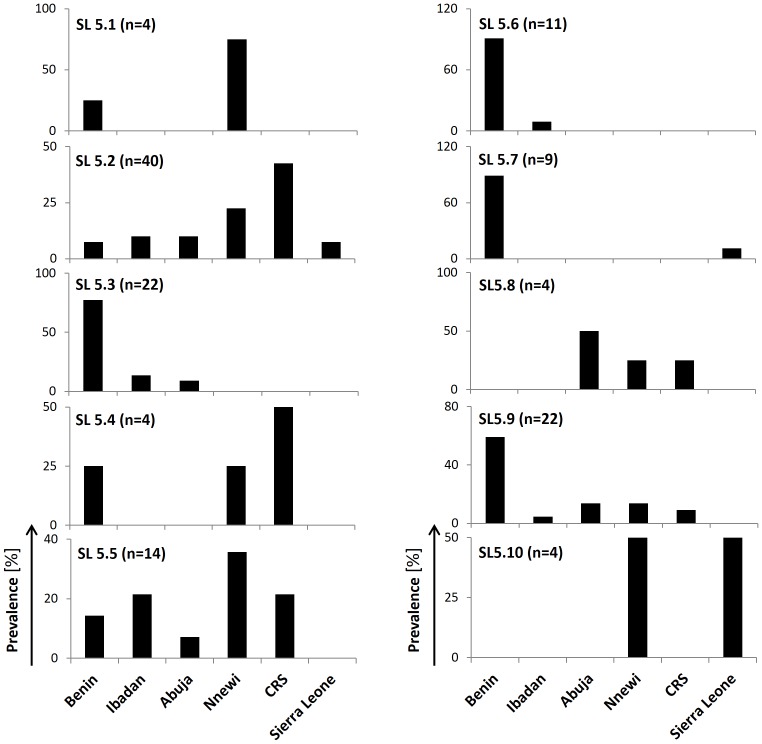
Geographical distribution of identified *M. africanum* West Africa 1 (MAF1) sub-lineages. (CRS = Cross River State).

We correlated the geographical origin of the strains with the obtained population structure and found a strong local restriction for some of the sub-lineages (see Figure 3). For instance, the majority (77–91%) of strains belonging to SL5.3, SL5.6 and SL5.7 could only be found in Benin. Similarly, SL5.9 mainly derives from Benin. In general, Benin showed the greatest genetic variety of strains, as 8 out of 10 sub-lineages were found there, with SL 5.8 and SL5.10 not identified in the present analysis. Conversely, sub-lineages with the lowest prevalence in Benin are the most frequent sub-lineages identified in Nigeria. For example, SL5.2 and 5.4 are mainly predominant in the Cross River State, with a minor share in Benin. Confirming this, SL5.1, SL5.5 and SL5.10 are the main sub-lineages in Nnewi and rare or even missing from Benin. Due to the small sample size of Sierra Leonean strains, it is difficult to describe geographic clustering, although all the analyzed isolates belonged to the three sub-lineages SL5.2, SL5.7 and SL5.10.

### Transmission Dynamics of *M. africanum* West African 1 Sub-lineages

To understand whether the identified sub-lineages differ in their ability to spread within the human host population we calculated the Recent Transmission Index RTI_n-1_ for each of them (see table 1). Interestingly, we found that there were great differences between the SLs. While, for instance, more than 50% of SL5.2 strains were recently transmitted, half of all remaining SLs were exclusively composed of singleton strains and were therefore due to re-activation of disease. Other sub-lineages that showed the capacity to effectively transmit within the host population were SL5.3, SL5.5, SL5.9 and SL5.6 (in decreasing order).

**Table 1:** Transmission dynamics of *M. africanum* West African 1 sub-lineages.

Sub-Lineage	Total numberstrains	Number clusteredstrains	Number singletonstrains	Numberclusters	Recent Transmissionindex (RTI_n-1_)
**SL5.1**	4	0	4	0	0
**SL5.2**	40	32	8	11	0.52
**SL5.3**	22	10	12	2	0.36
**SL5.4**	4	0	4	0	0
**SL5.5**	14	8	6	3	0.35
**SL5.6**	11	2	9	1	0.09
**SL5.7**	9	0	9	0	0
**SL5.8**	4	0	4	0	0
**SL5.9**	22	9	13	3	0.27
**SL5.10**	4	0	4	0	0
**Non SL**	7	0	7	0	0

## Discussion

In the current publication we intended to determine the *M. africanum* prevalence in Benin, with a special focus on the genotypic composition of the MAF1 lineage. In 2010, de Jong *et al.* presented the first comprehensive review on *M. africanum* and described the geographical distribution of the MAF1 lineage, with the highest prevalence in Ivory Coast (14%), Ghana (21%), Benin (39%), Nigeria (11%) and Cameroon (11%) [2]. Meanwhile more recent epidemiological surveys were conducted and observed a dramatic drop of MAF1 and MAF2 numbers in several West African countries [8], [17], [18], [19], suggesting that *M. africanum* is replaced by other lineages. In sharp contrast, two recent studies from several regions in Nigeria (Abuja, Ibadan, Nnewi and Cross River State) estimated persistently high MAF1 prevalence between 14% and 33% and detected foci of recent transmission [9], [10].

Based on manual interpretation of spoligotype patterns, the prevalence of MAF1 and MAF2 in Cotonou/Benin in 2005/06 was estimated to be 39% and 9%, respectively [2]. However, as manual interpretation of spoligotype data has limitations for lineage classification, we revisited the data from Affolabi *et al.* [11] and, first of all, separated *M. tuberculosis* strains from *M. africanum* using an automated online platform “TBLineage” for standardized lineage assignment to spoligotypes, which was not available before [13], [14], [15]. Subsequently, we confirmed the correct lineage assignment of all designated *M. africanum* strains with 12-loci MIRU-VNTR and determined the MAF1 and MAF2 prevalence to be 31% and 6%, respectively. Although the combination of spoligotyping and 12-loci-MIRU-VNTR was recently shown to correctly determine all 7 lineages of the MTBC [20], two of our isolates could not be unambiguously identified as MAF and were excluded from the study. If DNA were still available, we could have tested these isolates for presence or absence of lineage-defining region of differences RD7–10 [21], [22]. Nevertheless, our finding makes Benin the country with the second highest *M. africanum* and, together with the Cross River State, the highest MAF1 prevalence in the world.

Since infections with MAF1 are of such importance for the country and the region, we especially focused on the first genotypic analysis of circulating MAF1 strains from the sub-region. A recent publication established a robust global diversity of the MTBC and correctly identified the major MTBC lineages using a combination of spoligotyping and 12-loci-MIRU-VNTR genotyping data [20]. Therefore, we used the same genotyping methods to identify MAF1 and MAF2 strains. To put our MAF1 isolates in phylogenetic context, we constructed for the first time the population structure based on spoligo- and 12-loci MIRU-VNTR-typing data. Besides our strains from Benin, we included all publicly available genotypic strain information from Nigeria [9], [10] and Sierra Leone [12]. We found an unexpected great variability within the MAF1 lineage and we suggest the presence of at least ten sub-lineages (SL) within the MAF1 lineage. As MAF1 is also known as Lineage 5 [1], we designated the sub-lineages as SL5.1 to SL5.10. Interestingly, SL5.9 could potentially be further subdivided into four families. We also identified 7 isolates that could not have been assigned to any of the sub-lineages. This could be due to the fact that these isolates might be members of six further sub-lineages that would emerge if sample size was to be increased.

When correlating the geographical origins with the sub-lineage assignment of the population structure, we detected a high degree of geographical clustering for most of the sub-lineages. For instance, SL5.3, SL5.6 and SL5.7 are almost exclusively present in Benin. Similarly, almost 60% of all isolates of SL5.9 originated from Benin. Benin is also home to the largest genetic variability of MAF1 strains as 8 out of 10 sub-lineages were detected there. Interestingly, there appears to be a gradient between Benin and Nigeria as sub-lineages SL5.2, SL5.4, SL5.5, SL5.8 and SL5.10, which are mainly present in distant Cross River State and Nnewi, are either absent or the least frequent in Cotonou/Benin. Such a geographical gradient between the two areas could also explain the two foci with the highest MAF1 prevalence estimates of 31% (this study) and 33% [10] in Benin and Cross River State, respectively. Consistently, Ibadan (17%) and Nnewi (14%) both show medium prevalence, while in Abuja, at greater distance from Cotonou and the Cross River State, only 8% of all tuberculosis was due to MAF1 infection [9].

However, such geographical clustering was not observed for MAF1 strains from Sierra Leone. This could be due to the small numbers of strains included in our analysis, yet also suggests that MAF1 was not endemic in Sierra Leone before. Therefore it is possible that these strains were recently introduced into the country. From this analysis we hypothesize that the Sierra Leonean isolates in SL5.7 could have originated in Benin. Similarly, two other SL5.10 isolates from Sierra Leone most likely derive from Nnewi, where this particular sub-lineage is most common. The remaining 3 isolates from Sierra Leone were classified to be SL5.2, a lineage mainly present in the Cross River State and Nnewi. However, since for none of the studies the geographical sampling area was measured, we could not adjust for the number of studied isolates per geographical area of collection. Therefore there is the possibility, that the observed geographical differences are an artefact.

Besides the observed geographical restriction of the strains we were also interested whether the sub-lineages show differences in their capacity to infect the human host population. Based on the assumption that genotypically identical isolates belong to a chain of transmission [16] we calculated the Recent Transmission Indices RTI_n-1_ and found that some of the sub-lineages were superior in transmitting from human to human. This is especially intriguing as it might indicate an underlying genetic determinant responsible for effective transmission that certain sub-lineages might have and others lack. In particular, this wide range of spreading capacity within MAF1 makes this lineage a good model to study the phenomenon of variation in transmission in the future. Therefore, it would be very interesting to longitudinally follow up on our prediction and confirm whether the identified highly transmissible sub-lineages will indeed replace the slowly spreading ones over time. Taken together, we demonstrate that there are active foci of transmission in Eastern West Africa and that MAF1 persists as an important pathogen in the sub-region.

The presently included studies ranged from cross-sectional studies to convenience samples, and differed in study duration. Incomplete sampling and limited study duration tend to lead to an underestimation of the proportion of TB disease due to recent transmission [23], and constitutes a limitation of this study. Although the RTI_n-1_ intrinsically adjusts for the total number of sampled strains, such a scenario could be conceivable for SLs with smaller total number of strains, and therefore their RTI_n-1_ should be interpreted cautiously. However, we have no indication that this underestimation would differ between sub-lineages of larger number of total strains, and expect that the observed differences in the efficiency of transmission may be repeated in future population based studies. Confirming this is the fact that SL5.3 and SL5.9 have both identical total number of strains (n = 22), yet seem to transmit differently, reflected by an RTI_n-1_ of 0.36 and 0.27, respectively.

In the present publication, we identified Cotonou/Benin as one of the epicenters with the highest MAF1 prevalence in West Africa. Although MAF1 seems to disappear from certain regions in the area, it remains a major public health problem in others. Furthermore, we established the first comprehensive population structure for this lineage and were able to describe an unexpected genetic variability. Our analysis suggests the presence of at least 10 sub-lineages, that not only cluster based on genotypic data, but also geographically. These findings, however, would need to be conclusively confirmed in the future through whole genome sequencing and the construction of a SNP-based phylogeny.
